# Capillary Malformation–Arteriovenous Malformation Combined Alagille Syndrome in a Patient With Double Gene Variations of *RASA1* and *NOTCH2*

**DOI:** 10.3389/fgene.2019.01088

**Published:** 2019-11-05

**Authors:** Yu Zheng, Yuming Peng, Shuju Zhang, Liping Li, Yu Peng, Qiang Yin

**Affiliations:** ^1^Pediatrics Research Institute of Hunan Province, Hunan Children’s Hospital, Changsha, China; ^2^Research Center for Medical Genetics, School of Life Sciences, Central South University, Changsha, China; ^3^First Department of General Surgery, Hunan Children’s Hospital, Changsha, China

**Keywords:** vascular malformation, Abernethy malformation, Alagille syndrome, double gene variations, congenital heart disease, port-wine stain, liver

## Abstract

**Background:** Capillary malformation–arteriovenous malformation (CM-AVM) is an autosomal dominant disorder characterized by CMs, often in association with fast-flow vascular malformations. Alagille syndrome is an autosomal dominant multisystem disorder, usually involving hepatic, cardiac, ophthalmic, skeletal, or renal dysplasia. The combination of CM-AVM and Alagille syndrome in a patient presenting serious vascular malformations in the liver and heart has never been reported. Here, we report the case of a 20-month-old infant presenting these two diseases.

**Case presentation:** The patient manifested port-wine stains, congenital heart disease, cholestasis with abnormal morphology, and vascular anomalies. Color Doppler (B-mode) ultrasonography, and radiological imaging including computed tomography (CT) with enhanced three-dimensional (3D) reconstruction and angiography, revealed a type II Abernethy malformation in the hepatic portal vein. The left hepatic lobe was enlarged showing dilation of the portal vein and the left artery. Whole exome sequencing (WES) identified a paternally inherited *RASA1* heterozygous pathogenic variant p.(Ser219Ter) causing CM-AVM and a *de novo NOTCH2* heterozygous variant p.(Met2042Thr) associated with Alagille syndrome.

**Conclusion:** This is the first case of combined CM-AVM and Alagille syndrome presenting serious liver and heart abnormalities diagnosed using imaging technology and WES. The patient harbored variants in two genes: *RASA1* and *NOTCH2*, which rarely contribute to aberrant vascular development. This report highlights the value of accurately diagnosing similar diseases and guiding therapy using genetic testing combined with careful clinical examinations.

## Background

CM-AVM (OMIM: 608354) is characterized by atypical capillary malformations (CMs) and often accompanied by multiple arteriovenous malformations (AVMs), at least one fast-flow vascular anomaly, arteriovenous fistulas (AVFs), or Parkes Weber syndrome (PWS) (Eerola et al., 2003; Boon et al., 2005; Revencu et al., 2008). CMs are commonly manifested by cutaneous multifocal port-wine stains (also referred as nevus flammeus, stork bite, nevus simplex, or flaky purplish red hemangiomas) (Eerola et al., 2003; Boon et al., 2005). PWS can present large cutaneous vascular stains, multiple AVFs, and overgrowth of affected limbs (Revencu et al., 2008). It is an autosomal dominant disorder and the associated gene is *RASA1* (Eerola et al., 2003). Heterozygous inactivating *RASA1* variants could lead to the phenotypic variability of CM-AVM (Revencu et al., 2013). RASA1 is related to the p120-RasGAP signaling pathway, which is activated by a variety of growth factor receptors and regulates the proliferation, migration, and survival of vascular endothelial cells and some other cell types (Eerola et al., 2003).

Alagille syndrome (OMIM: 610205) is a rare congenital disease with multisystem clinical features involving the liver, cardiac, facial, skeletal, and ocular abnormalities (Krantz et al., 1997; Turnpenny and Ellard, 2012; M and KM, 2018). Liver synthetic dysfunction commonly presents as chronic cholestasis (seen in ~89% of patients) (Subramaniam et al., 2011). Intrahepatic bile duct paucity is seen in ~75% of patients (Subramaniam et al., 2011). Cardiac abnormalities are mainly associated with congenital heart disease (seen in up to 94% of patients) and most frequently with peripheral pulmonary artery stenosis, and may include atrial and/or ventricular septal defects and patent ductus arteriosus (M and KM, 2018). Some cases manifest dysmorphic facies with a broad forehead, deep-set eyes, and pointed chin. A few cases present skeletal anomalies with “butterfly” vertebrae (~ 50% of cases) and some cases present with tapering distal phalanges. Ophthalmic anomalies with anterior chamber defects may also occur (Subramaniam et al., 2011; Turnpenny and Ellard, 2012). Renal, vascular, neurodevelopmental, or growth anomalies are also present in some cases (Turnpenny and Ellard, 2012). Alagille syndrome is an autosomal dominant disorder caused by defects in the Notch signaling pathway, and *JAG1* and *NOTCH2* are the two major related genes (Li et al., 1997; Oda et al., 1997; McDaniell et al., 2006; M and KM, 2018). The phenotypic penetrance and expressivity may be variable in the differently affected individuals (M and KM, 2018).

To our knowledge, the combination of CM-AVM and Alagille syndrome in one patient has not been previously reported. Here, we present the case of a 20-month-old girl who presented serious portal vein and artery malformations in the liver and heart, as revealed by enhanced CT, angiography, and ultrasonography inspections. She suffered cholestasis and congenital heart disease. Whole exome sequencing (WES) detected two germline pathogenic variants in *RASA1* and *NOTCH2* genes and further confirmed that she had these two syndromes.

## Case Presentation

**Figure 1:**
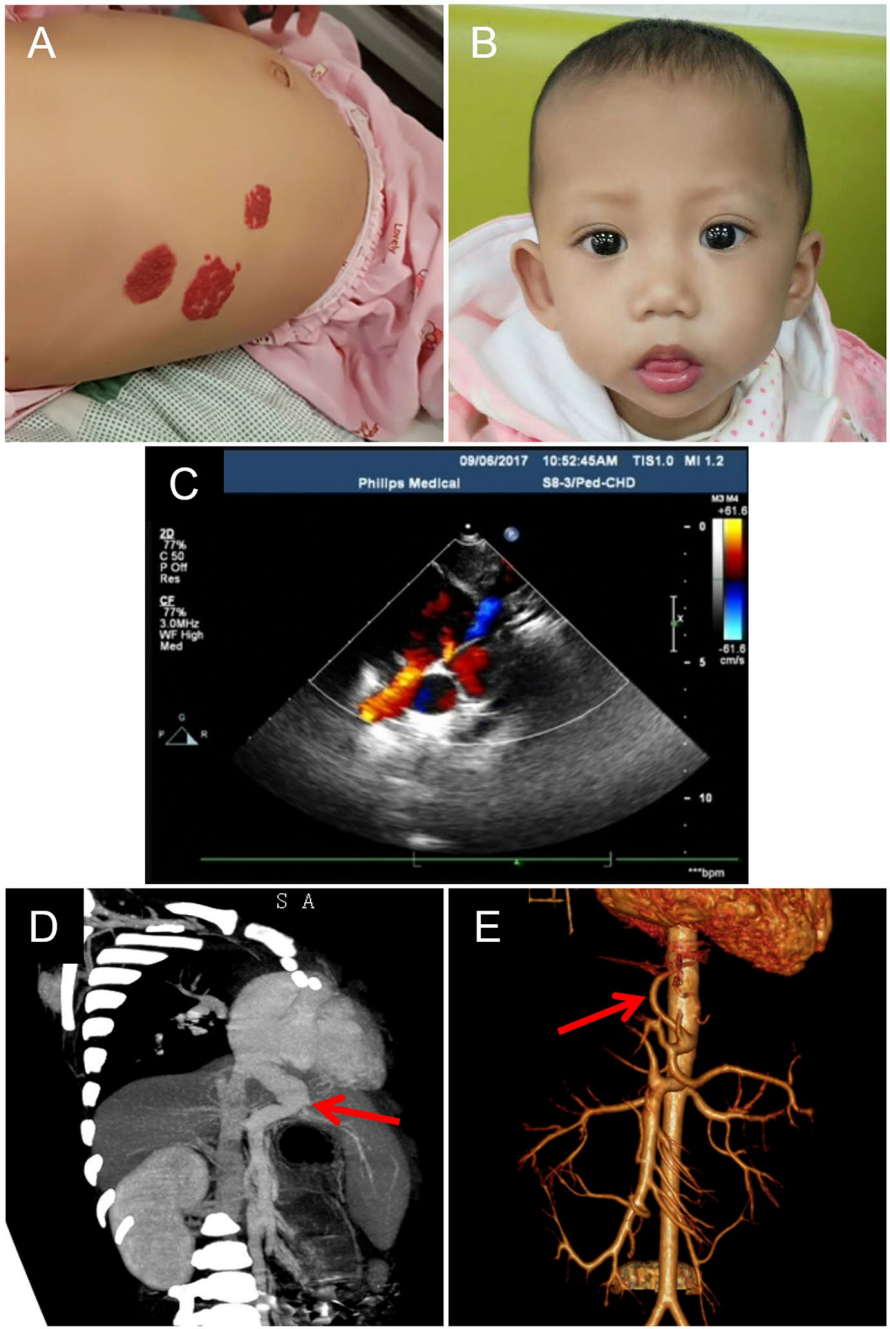
Skin capillary malformations, facial feature, and heart and liver arteriovenous malformations of the patient. **(A)** Multiple flaky purplish red hemangiomas presented in the abdomen. **(B)** The characterized face of the patient shows a broad forehead, a small-pointed chin, and prominent ears. **(C)** The image of color Doppler b mode ultrasonography shows congenital heart disease: patent ductus arteriosus, patent foramen ovale, tricuspid, and mitral regurgitation. **(D)** The CT image shows enlarged portal vein draining directly into the inferior vena cava (red arrow). **(E)** Angiography shows enlarged left hepatic artery (red arrow).

A female infant with a history of neonatal septicemia, multiple osteomyelitis, and furuncle swelling of the skin and soft tissue was admitted to Hunan Children’s Hospital (Hunan Province, China). When she was 1 month old, several AVMs were revealed in her liver and skin. Multiple nevus flammeus protruded from her skin in the area of thorax, abdomen, and legs for more than 1 year (**Figure 1A**). Her father also presented several nevus flammeus. Her face was characterized by a broad forehead, a small-pointed chin, and prominent ears (**Figure 1B**). When she was 10 months old, color Doppler (B-mode) ultrasonography revealed an enlarged right heart, patent ductus arteriosus (1.5 mm), severe tricuspid regurgitation, and pulmonary arterial hypertension (TR Vmax = 435 cm/s, SPAP = 76 mm Hg) (**Figure 1C**). The aorta and atrium were right-to-left shunted horizontally. Bilateral cardiac catheterization was performed to improve her heart condition.

At the age of 20 months, severe hepatic blood vessel malformations due to cholestatic hepatitis and pneumonia were revealed using radiological imaging technology including computed tomography (CT) with enhanced three-dimensional (3D) reconstruction and angiography. The left lobe of her liver was enlarged (72 × 59 mm), and the right lobe of the liver was small (54 × 55 mm). The portal vein was dilated and directly connected to the inferior vena cava (**Figure 1D**), and then drained to the right atrium. The intrahepatic portal vein (the left branch) in the left lobe was small, and the right branch in the right lobe was unclear. This malformation of the portal vein was characterized as type II Abernethy malformation. The left hepatic artery (~2.6 mm, Vmax = 34 cm/s) was dilated (**Figure 1E**) and circuitous, and the right hepatic artery was small (1.8 mm). Imaging also revealed small ductus arteriosus and stenosis of the left main bronchus. The left main bronchus was narrow, focally. Either intrahepatic or extrahepatic bile ducts showed no obvious dilation. The liver function tests showed synthetic dysfunction: total bilirubin 28.6 µmol/L, direct bilirubin 9.2 µmol/L, indirect bilirubin 19.4 µmol/L, total bile acid 62.9 µmol/L, alanine aminotransferase 22.3 IU/L, and aspartate aminotransferase 46.7 IU/L. The patient had no family history of hepatitis.

**Figure 2:**
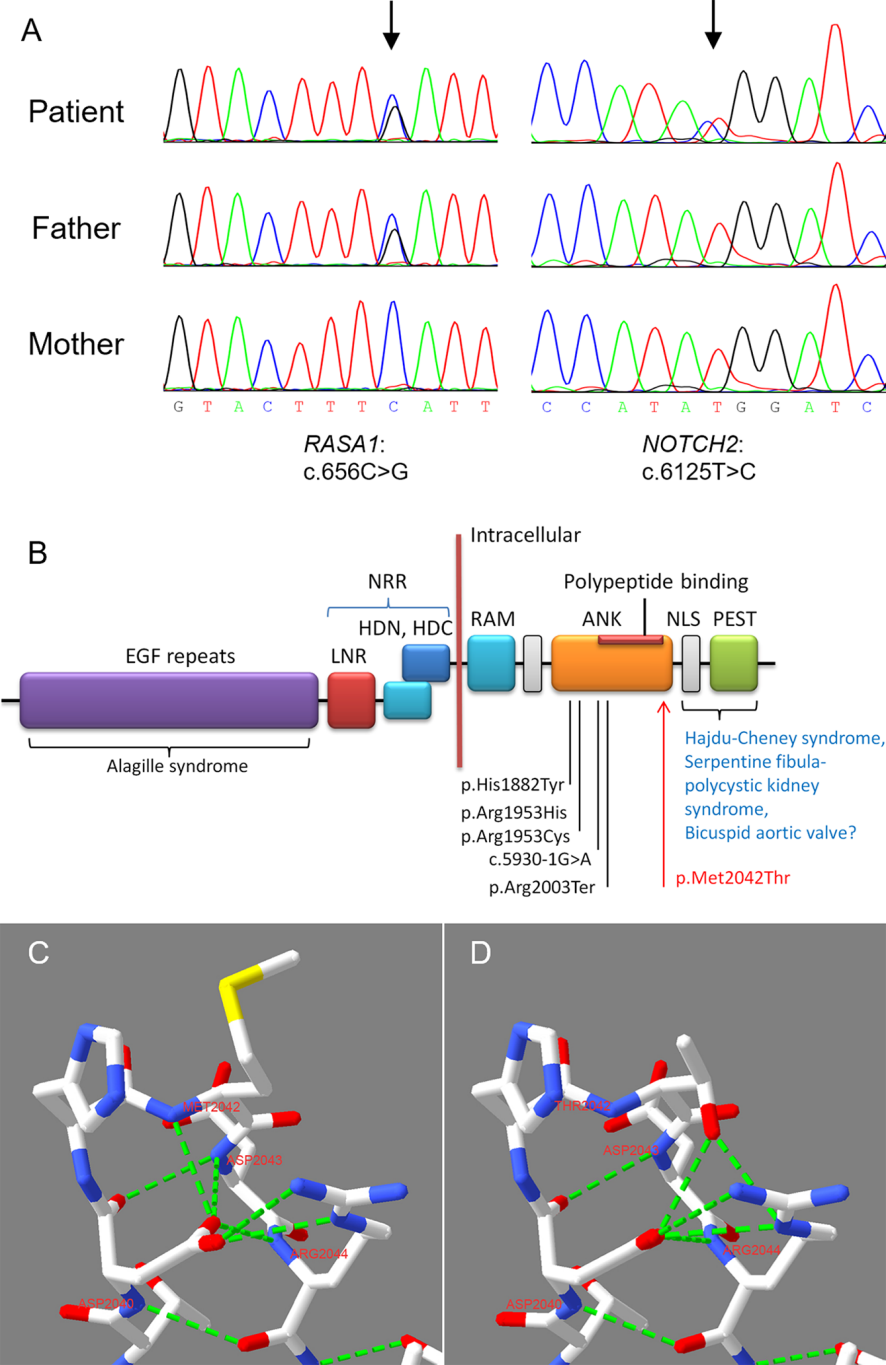
Sanger sequencing verified the two detected variants and homology modeling of the *NOTCH2* variant. **(A)** Sanger sequencing of the trios confirmed the *RASA1* and *NOTCH2* variants. **(B)** NOTCH2 domain structure and the locations of pathogenic variants associated with Alagille syndrome and other diseases. The known pathogenic variants in ANK domain associated with Alagille syndrome were marked. Our reported p.Met2042Thr in this case is highlighted using red arrow. EGF Repeats, epidermal growth factor-like repeats; ANK, ankyrin repeats; LNR, Lin/Notch repeats; NRR, negative regulatory region, including the two heterodimeric portions (HDN and HDC) interact and LNRs; NLS, nuclear localization signal; PEST, proline/glutamic acid/serine/threonine rich domain; RAM, RBP-Jκ-associated module (Kamath et al., 2012; Bray, 2016). **(C)** In the WT ANK domain, Asp2040 formed two strong H-bonds with Met2042 and Asp2043. **(D)** In the mutant ANK domain, two normal H-bongs formed by Asp2040 were damaged, then Thr2042 formed two excrescent strong H-bonds with Asp2040 and Arg2044, respectively. Red represents O, blue represents N, and ﬂuorescent green represents strong H-bond.

Genetic testing using WES was performed to identify any pathogenic variants. Genomic DNA was isolated from peripheral blood leukocytes of the patient and her parents. DNA samples were sheared, and libraries were prepared to capture the coding sequences of human coding genes using xGEN Exome Research Panel v1.0 (Integrated DNA Technologies, Coralville, USA) following the manufacturer’s protocol. The captured libraries were then clustered and sequencing was performed on the Illumina HiSeq X Ten system (Illumina, San Diego, California, USA) to generate 2×150 bp reads. Each sample yielded over 11.2 Gb raw data. Over 89% (average ~ 92.0%) of bases had Phred quality score > 30. After removing the low-quality reads and adapter-contaminated reads, the data were aligned to the human reference genome (version: Hg19) using NovoAlign software (http://www.novocraft.com/products/novoalign/). Alamut (https://www.interactive-biosoftware.com/alamut-visual/) was then employed to annotate each variant. We used Varscan (version 2.3.8, http://varscan.sourceforge.net/) software to call SNPs and InDels. We removed benign or likely benign variants according to the American College of Medical Genetics and Genomics (ACMG) criteria (Richards et al., 2015) and retained pathogenic or likely pathogenic variants at a high priority. After the bioinformatics analysis, we detected a heterozygous nonsense variant in *RASA1* [NM_002890.2:c.656C > G, NP_002881.1:p.(Ser219Ter)] in the patient and her father (**Table 1**). In addition, a *de novo* heterozygous missense variant in *NOTCH2* [NM_024408.3:c.6125T > C, NP_077719.2:p.(Met2042Thr)] was identified only in the patient (**Figure 2A**, **Table 1**). We further performed Sanger sequencing in the two targeted variant sites *via* PCR-based exon amplification and direct bidirectional sequencing. The two variants were validated in the patient, and her father carried *RASA1* p.(Ser219Ter). Her mother had a wild-type genotype (**Figure 2A**). The sequence of the novel variant *NOTCH2* p.(Met2042Thr) in the ankyrin repeats (ANK) domain was loaded to SWISS-MODEL (http://swissmodel.expasy.org/) for homology modeling. Swiss-PdbViewer (SPDBV) software was used to analyze the structural changes of the mutant AT protein and generate structural figures.

**Table 1:** Information on the two variants identified in the patient.

Gene	NOTCH2	RASA1
Nucleotide Change	NM_024408.3:c.6125T > C	NM_002890.2:c.656C > G
Amino Acid Change	NP_077719.2:p.(Met2042Thr)	NP_002881.1:p.(Ser219Ter)
ACMG Criteria	Pathogenic (PS2, PM1, PM2, PP2, PP3)	Pathogenic (PVS1, PM2, PP3, PP4, PP5)
Novel/Known Variation	Novel	ClinVar collected
Inheritance Mode	*De novo*	Paternal
Frequency in Cohorts (GnomAD, 1000 genome, ESP6500, ExAC)	0	0
GERP++_RS^#^	Conserved	Conserved
CADD_pred^&^	Damaging	Damaging
SIFT_phred^?^	Damaging	Damaging
DANN_pred^§^	Damaging	Damaging
VEST3_pred*	Damaging	Damaging

*^#^*Predicted conservative property by Genome Evolutionary Rate Profiling (GERP) version 2 (Davydov et al., 2010).

^&^Predicted pathogenicity by Combined Annotation Dependent Depletion (Rentzsch et al., 2019).

*^※^*Predicted pathogenicity by Sort intolerated from tolerated (SIFT) (Ng and Henikoff, 2003).

^§^Predicted pathogenicity by Deleterious Annotation of genetic variants using Neural Networks (DANN) (Quang et al., 2015).

* Predicted pathogenicity by Variant effect scoring tool (VEST) version 3 (Carter et al., 2013).

The two variants were pathogenic according to the ACMG criteria (**Table 1**) (Richards et al., 2015). The heterozygous nonsense variant p.(Ser219Ter) in *RASA1* has been reported in a family with a CM (Revencu et al., 2008), and was recorded in the Human Gene Mutation Database (HGMD, http://www.hgmd.cf.ac.uk) and ClinVar (https://www.ncbi.nlm.nih.gov/clinvar/variation/464870/) databases. The *NOTCH2* variant p.(Met2042Thr) was novel. It was located in the ANK domain of the Notch intracellular region (**Figure 2B**), which was highly conserved (GERP++_RS score = 5.84) and was classified as pathogenic according to the ACMG criteria. The variant was adjacent to the polypeptide binding region in the ANK domain (**Figure 2B**). Homology modeling and structural analysis of the p.(Met2042Thr) variation suggested that in the ANK domain of the wild-type NOTCH protein, the side chain of Asp2040 formed two strong H-bonds with the main chain amides of Met2042 and Asp2043 (**Figure 2C**). When the Met2042 changed into Thr2042, these two H-bonds did not occur, and the side chain of Thr2042 formed two excrescent strong H-bonds with Asp2040 and Arg2044, respectively (**Figure 2D**). Heterozygous variants in *NOTCH2*, with a high majority of missense variants, are known to cause Alagille syndrome (M and KM, 2018). The patient had a broad forehead, a small-pointed chin and prominent ears. With the addition of congenital heart disease and cholestasis, her phenotypes conformed to the diagnosis of Alagille syndrome (Turnpenny and Ellard, 2012). Thus, genetic testing combined with clinical features confirmed Alagille syndrome and CM-AVM in this patient. The patient died while waiting for liver transplantation.

## Discussion

Both CM-AVM and Alagille syndrome are rare diseases with a wide variety of clinical features and manifestations. It is difficult to diagnose the two diseases using limited clinical phenotypes only. Particularly, when the two diseases coexist in one person, the clinical features might be more complex, and it will be harder to make an accurate diagnosis. In the case reported herein, the patient had various manifestations in the liver, heart, skin, facies, and pulmonary bronchus. We applied a 3D reconstruction of the blood vessels, which was able to reveal extrahepatic portocaval shunts. We also performed CT and magnetic resonance (MRI) in addition to routine inspections, and successfully identified clinical features thoroughly. To further identify the disease-causing factors, genetic testing was performed to accurately diagnose the disease and guide therapy. Considering both her phenotype and genotype, she was diagnosed with CM-AVM and Alagille syndrome. Having pathogenic variants in two disease-causing genes *RASA1* and *NOTCH2*, she developed type II Abernethy malformation and other vascular anomalies in the liver and heart.

CM-AVM is a congenital vascular malformation that may involve capillary, vein, artery, lymph-vessel, or combined anomalies (Revencu et al., 2013). It presents at birth and usually occurs as a sporadic and isolated lesion. Some cases are familial, and some are part of a syndrome, such as Parkes Weber syndrome, Sturge-Weber syndrome, CLOVES syndrome, and hereditary hemorrhagic telangiectasia (Bloom and Upton, 2013; Comi, 2015; Shovlin, 2010; Banzic et al., 2017). CM-AVM is attributed to heterozygous mutations in *RASA1*. In addition to CM, fast-flow lesions frequently happen in CM-AVM. No hepatic or pulmonary AVMs or AVFs have been found in over 300 patients harboring an *RASA1* variant, as presented in a previous study (Revencu et al., 2013). In this case, the *RASA1* variant p.(Ser219Ter) was inherited from her father. Her father was 29 years old and only manifested CM with multiple nevus flammeus. No other malformations were found. This variant was already recorded in ClinVar (ID: 464870) with CM-AVM and/or basal cell carcinoma phenotypes.

Patients with Alagille syndrome are initially diagnosed based on intrahepatic bile duct paucity and other three or more of the five clinical features: cholestasis, dysmorphic facies, congenital heart disease, vertebral anomalies, and ocular abnormalities (Turnpenny and Ellard, 2012). The patients also have a high frequency of renal and vascular anomalies (M and KM, 2018). It has been suggested that vasculopathy is the major feature of Alagille syndrome. Since the formation of the intrahepatic arterial branch affects the development of intrahepatic bile ducts, it may cause bile duct paucity (M and KM, 2018). However, no obvious vascular malformations in the liver have been observed in Alagille patients, particularly portal vein anomalies. This is the first report of a patient with Alagille syndrome presenting with considerable vascular malformations in the liver.

Whether the particular liver aberrations in morphology or blood vessels are associated with both *NOTCH2* and *RASA1* variants is unclear. In this study, this patient manifested both Type II Abernethy malformation and abnormal hepatic artery. She presented cholestasis and a *de novo* missense variant in *NOTCH2* was identified. This variant was novel and is proposed to cause Alagille syndrome. Gilbert M. et al. have concluded that almost 77% of *NOTCH2* variants were missense variants in the screened Alagille syndrome patients (Stenson et al., 2017; M and KM, 2018). Our identified variant, p.(Met2042Thr), was located in the intracellular ANK repeats of Notch 2, which is involved in the interaction with transcription factors (Li et al., 2006; Wilson and Kovall, 2006). Therefore, this variant may affect *NOTCH2* gene expression and Notch signaling (20). *RASA1* encodes the RAS GTPase activating protein, which negatively regulates the RAS-MAPK signal pathway. Both RAS-MAPK and Notch signaling pathways are involved in the vascular development (Chen et al., 2019; Serrano and Demarest, 2019). In addition, a single variant in *NOTCH2* or *RASA1* may cause phenotypes associated with vascular malformations. Therefore, combining the effect of variants of these two genes probably causes much more serious and complicated vascular malformations than one single variant.

In these two syndromes, the particular hepatic vascular anomalies have rarely been reported so far, and the underlying pathogenesis needs to be further studied. On one hand, it is important to examine the effect of the novel *NOTCH2* variant on RNA and protein expression. On the other hand, somatic mutations arising in the *RASA1* gene in specific organs could cause focal lesions. In some of the CM-AVM cases, somatic second hit mutations have been found in other locus of *RASA1* in the vascular endothelial cells or the focal tissue (Revencu et al., 2013; Cai et al., 2018; Lapinski et al., 2018; Ten Broek et al., 2019). A genetic analysis of *RASA1* in hepatic tissue could help to confirm this. The mechanism by which the *NOTCH2* variant together with the *RASA1* variant leads to vascular developmental malformations in liver and heart remains to be further investigated.

## Conclusions

To our knowledge, this is the first reported case of both *RASA1* and *NOTCH2* pathogenic variants in CM-AVM, Alagille syndrome, and Abernethy malformation. The combination of examinations, such as CT and 3D reconstruction of blood vessels, and exome sequencing may provide an accurate diagnosis. We identified a novel *NOTCH2* missense variant, which enriched the human variant database underlying Alagille syndrome. Together with the known *RASA1* pathogenic variant, they contributed to severe vascular anomalies in the liver, heart, and cutaneous CMs, which have never been previously reported.

## Data Availability Statement

The raw data supporting the conclusions of this manuscript will be made available by the authors, without undue reservation, to any qualified researcher.

## EthicS Statement

The samples were obtained with appropriate informed consent from all participants.

## Author Contributions

QY: supervision and resources acquisition. YP: methodology, validation, and data visualization. YZ: original manuscript writing and editing, data analysis. YMP: sample collection and clinical data curation and validation. SZ and LL: methodology and resources collection. YP, QY, and YMP: manuscript review and editing. All authors read and approved the final manuscript.

## Funding

Health Commission of Hunan province, Grant/Award Number: the technological innovation project of the key specialty development “Liver transplantation for biliary atresia in children” in Hunan Children’s Hospital. Hunan Children’s Hospital, Grant/Award Number: Key project of 2018 “Bioinformatics pipeline for pediatrics genetic diseases.”

## Conflict of Interest

The authors declare that the research was conducted in the absence of any commercial or financial relationships that could be construed as a potential conflict of interest.

## Abbreviations

CM, capillary malformation; AVM, arteriovenous malformation; WES, whole exome sequencing; CT, computed tomography; PWS, Parkes Weber syndrome; ACMG, American College of Medical Genetics and Genomics; HGMD, the Human Gene Mutation Database; AVFs, arteriovenous fistulas; ANK, ankyrin repeats.
